# Safety and Pharmacokinetic Characterization of Nacubactam, a Novel β-Lactamase Inhibitor, Alone and in Combination with Meropenem, in Healthy Volunteers

**DOI:** 10.1128/AAC.02229-19

**Published:** 2020-04-21

**Authors:** Navita L. Mallalieu, Erica Winter, Scott Fettner, Katie Patel, Elke Zwanziger, Gemma Attley, Ignacio Rodriguez, Akiko Kano, Sameeh M. Salama, Darren Bentley, Anna Maria Geretti

**Affiliations:** a Roche Innovation Center, New York, New York, USA; b Roche Innovation Center, Welwyn, United Kingdom; c Roche Innovation Center, Basel, Switzerland; d Meiji Seika Pharma Co., Ltd., Tokyo, Japan; e Fedora Pharmaceuticals, Inc., Edmonton, Alberta, Canada; f Institute of Infection and Global Health, University of Liverpool, Liverpool, United Kingdom

**Keywords:** beta-lactam, beta-lactamase inhibitor, meropenem, multiple ascending dose, nacubactam, pharmacokinetics, phase I, single ascending dose

## Abstract

Nacubactam is a novel β-lactamase inhibitor with dual mechanisms of action as an inhibitor of serine β-lactamases (classes A and C and some class D) and an inhibitor of penicillin binding protein 2 in *Enterobacteriaceae*. The safety, tolerability, and pharmacokinetics of intravenous nacubactam were evaluated in single- and multiple-ascending-dose, placebo-controlled studies. Healthy participants received single ascending doses of nacubactam of 50 to 8,000 mg, multiple ascending doses of nacubactam of 1,000 to 4,000 mg every 8 h (q8h) for up to 7 days, or nacubactam of 2,000 mg plus meropenem of 2,000 mg q8h for 6 days after a 3-day lead-in period. Nacubactam was generally well tolerated, with the most frequently reported adverse events (AEs) being mild to moderate complications associated with intravenous access and headache. There was no apparent relationship between drug dose and the pattern, incidence, or severity of AEs. No clinically relevant dose-related trends were observed in laboratory safety test results. No serious AEs, dose-limiting AEs, or deaths were reported. After single or multiple doses, nacubactam pharmacokinetics appeared linear, and exposure increased in an approximately dose-proportional manner across the dose range investigated. Nacubactam was excreted largely unchanged into urine. Coadministration of nacubactam with meropenem did not significantly alter the pharmacokinetics of either drug. These findings support the continued clinical development of nacubactam and demonstrate the suitability of meropenem as a potential β-lactam partner for nacubactam. (The studies described in this paper have been registered at ClinicalTrials.gov under NCT02134834 [single ascending dose study] and NCT02972255 [multiple ascending dose study].)

## Abstract (precis)

Nacubactam is a novel β-lactamase inhibitor with dual mechanisms of action as an inhibitor of serine β-lactamases (classes A and C and some class D) and an inhibitor of penicillin binding protein 2 in *Enterobacteriaceae*. The safety, tolerability, and pharmacokinetics of intravenous nacubactam were evaluated in single- and multiple-ascending-dose, placebo-controlled studies. Healthy participants received single ascending doses of nacubactam of 50 to 8,000 mg, multiple ascending doses of nacubactam of 1,000 to 4,000 mg every 8 h (q8h) for up to 7 days, or nacubactam of 2,000 mg plus meropenem of 2,000 mg q8h for 6 days after a 3-day lead-in period.

## INTRODUCTION

Nacubactam (OP0595, RG6080) is a novel β-lactamase inhibitor with a distinctive dual mechanism of action. It is an inhibitor of bacterial serine β-lactamases (classes A and C and some class D) that prevents inactivation by hydrolysis of coadministered β-lactam agents. This protective mechanism is comparable with other molecules in the same class (e.g., avibactam). However, nacubactam is also an inhibitor of penicillin binding protein 2 (PBP2) in the cell wall of *Enterobacteriaceae*, exerting direct antibacterial effects and enhancing the activity of coadministered β-lactam agents (1). *In vitro* and *in vivo* studies showed that nacubactam combined with β-lactam antibiotics was active against bacteria producing any of the four classes of β-lactamases, including metallo-β-lactamases (class B) and oxacillinase (class D) (1–3). Nacubactam is being developed in combination with the β-lactam agent meropenem as a treatment for carbapenem-resistant *Enterobacteriaceae* infections.

Here, we report the results of single-ascending-dose (SAD) and multiple-ascending-dose (MAD), placebo-controlled studies, with the aim of assessing the safety, tolerability, and pharmacokinetics of nacubactam alone and in combination with meropenem. Additionally, these studies were intended to quantify potential pharmacokinetic drug-drug interactions between nacubactam and meropenem in healthy individuals.

## RESULTS

### Study participants.

**FIG 1:**
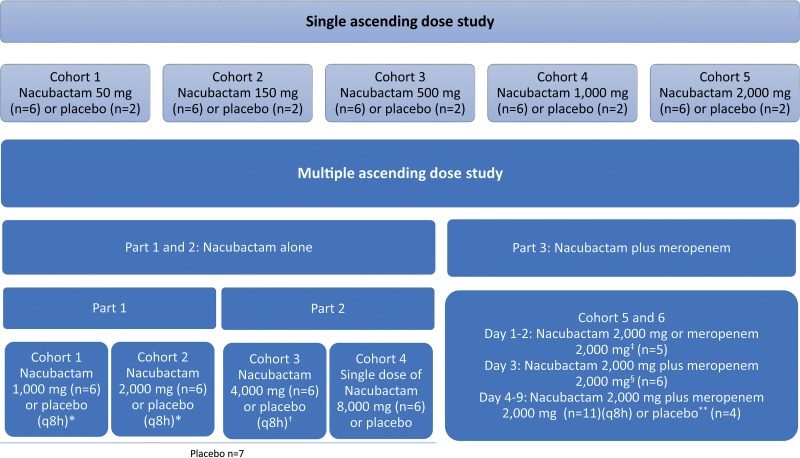
Overview of the SAD and MAD study designs. Symbols: *, dosing q8h for 7 days; †, single dose on day 1, with the option to extend to q8h dosing for 7 days after confirmation of pharmacokinetics, safety, and tolerability; ‡, single dose of either nacubactam or meropenem, allocated in a crossover manner on days 1 and 2; §, single dose of both drugs in combination; **, single dose on days 1 to 3. q8h, dosing on days 4 to 9. q8h, every 8 h.

A total of 40 healthy participants (all males aged 20 to 45 years, with eight participants per cohort randomized 6:2 to nacubactam or placebo) were enrolled in the SAD study, which evaluated intravenous (i.v.) doses up to 2,000 mg (Fig. 1). In the 3-part randomized, placebo-controlled MAD study, a total of 46 healthy participants (45 males and one female, aged 21 to 67 years) were enrolled to evaluate i.v. doses of up to 4,000 mg every 8 h (q8h) for up to 7 days (Fig. 1).

One participant from part 3 of the MAD study withdrew consent due to adverse events (AE) experienced after coadministration of nacubactam and meropenem (these AEs are described in full in “Safety and tolerability,” below). All other participants completed the study as planned, with no major protocol deviations. The baseline characteristics of participants who completed the study were balanced among treatment groups in both the SAD and MAD studies (Table 1). Mean baseline creatinine clearance was >113 ml/min in all participants, per eligibility criteria.

**TABLE 1:** Baseline characteristics of included participants^*a*^

Characteristic	SAD study (all male, Caucasian, non-Hispanic)						MAD study (parts 1 and 2)					MAD study (part 3)		
	Nacubactam, 50 mg (*n* = 6)	Nacubactam, 150 mg (*n* = 6)	Nacubactam, 500 mg (*n* = 6)	Nacubactam, 1,000 mg (*n* = 6)	Nacubactam, 2,000 mg (*n* = 6)	Placebo (*n* = 10)	Nacubactam, 1,000 mg (*n* = 6)	Nacubactam, 2,000 mg (*n* = 6)	Nacubactam, 4,000 mg (*n* = 6)	Nacubactam, 8,000 mg (single dose) (*n* = 6)	Placebo (*n* = 7)	Nacubactam→ meropenem→ combination (*n* = 5)	Meropenem→ nacubactam→ combination (*n* = 6)	Placebo (*n* = 4)
Mean (SD) age, yr	27.67 (3.98)	22.33 (1.97)	27.50 (9.35)	24.67 (3.01)	23.50 (3.73)	30.60 (7.12)								
Mean (SD) height, cm	181.43 (8.94)	179.50 (6.22)	180.25 (5.31)	175.53 (8.81)	179.35 (3.81)	179.36 (7.67)								
Mean (SD) wt, kg	83.03 (11.92)	77.02 (10.87)	82.90 (9.85)	72.83 (10.26)	76.28 (11.15)	79.12 (9.60)								
Mean (SD) BMI, kg/m^2^	25.10 (2.11)	23.80 (1.95)	25.40 (1.67)	23.58 (2.41)	23.62 (2.73)	24.56 (2.11)								
Mean (SD) age, yr							47.3 (15.3)	32.8 (12.3)	44.2 (16.7)	41.8 (13.8)	44.7 (16.0)			
Male, *n* (%)							6 (100.0)	6 (100.0)	5 (83.3)	6 (100.0)	7 (100.0)			
Race, *n* (%)														
Asian							0	0	0	0	1 (14.3)			
Black							1 (16.7)	4 (66.7)	2 (33.3)	2 (33.3)	3 (42.9)			
White							5 (83.3)	2 (33.3)	4 (66.7)	4 (66.7)	3 (42.9)			
Ethnicity, non-Hispanic, *n* (%)							6 (100.0)	6 (100.0)	6 (100.0)	5 (83.3)	7 (100.0)			
Mean (SD) height, cm							180.7 (4.4)	174.5 (6.8)	178.5 (7.9)	176.8 (5.2)	178.9 (7.0)			
Mean (SD) wt, kg							83.57 (8.18)	78.27 (9.61)	83.28 (11.81)	84.90 (5.62)	83.17 (6.15)			
Mean (SD) BMI, kg/m^2^							25.69 (3.27)	25.75 (3.20)	26.14 (3.24)	27.17 (1.78)	26.01 (1.55)			
Mean age (SD), yr												40.0 (12.3)	48.5 (13.7)	40.5 (14.6)
Male, *n* (%)												5 (100.0)	6 (100.0)	4 (100.0)
Race, *n* (%)														
Asian												0	0	1 (25.0)
Black												2 (40.0)	2 (33.3)	1 (25.0)
White												3 (60.0)	4 (66.7)	2 (50.0)
Ethnicity, non-Hispanic, *n* (%)												4 (80.0)	5 (83.3)	4 (100.0)
Mean (SD) height, cm												178.8 (2.8)	170.3 (6.0)	178.3 (3.3)
Mean (SD) wt, kg												81.42 (13.68)	81.07 (7.25)	83.65 (8.45)
Mean (SD) BMI, kg/m^2^												25.43 (3.94)	27.91 (1.54)	26.37 (3.05)

a BMI, body mass index; MAD, multiple ascending dose; SAD, single ascending dose; SD, standard deviation.

### Safety and tolerability.

All single doses of nacubactam up to 8,000 mg and multiple doses up to 4,000 mg q8h were well tolerated. Most AEs were mild, and all AEs resolved without sequelae. Only eight of the 30 (26.7%) participants reported one or more AEs, for a total of 11 AEs (see Table S1 in the supplemental material). In the placebo group, 4 of the 10 (40.0%) participants reported one or more AEs, for a total of five AEs.

In the MAD study, nacubactam alone or coadministered with meropenem was generally well tolerated. During the monotherapy phase (parts 1 and 2) of the MAD study, 13 of the 24 (54.2%) participants who received nacubactam reported a total of 48 AEs (Table 2), with the most frequently reported AEs being complications associated with i.v. access (medical device site erythema, injection site pain, catheter site pain, injection site erythema, injection site extravasation, injection site hemorrhage, injection site injury, and vessel puncture site hemorrhage) and headache. Of the seven participants who received placebo, three (42.9%) participants reported one AE each.

**TABLE 2:** Summary of TEAEs by treatment group in the MAD study

Parameter^*a*^	Parts 1 and 2					Part 3	
	Nacubactam, 1,000 mg (*n* = 6)	Nacubactam, 2,000 mg (*n* = 6)	Nacubactam, 4,000 mg (*n* = 6)	Nacubactam, 8,000 mg (single dose) (*n* = 6)	Placebo (*n* = 7)	Nacubactam + meropenem (*n* = 11)	Placebo (*n* = 4)
≥1 TEAE (*n*/E)	4/8	2/3	6/36	1/1	3/3	9/42	1/1
≥1 drug-related TEAE (*n*/E)	2/2	2/2	3/3	0	0	7/7	0
≥1 serious TEAE (*n*)	0	0	0	0	0	0	0
Withdrawn due to AE (*n*)	0	0	0	0	0	1	0
Specific TEAEs occurring in ≥2% of participants (*n*/E)							
Medical device site erythema^*b*^	0	0	4/4	0	0	0	0
Injection site pain	0	0	3/3	0	0	0	0
Catheter site pain	1/1	0	0	0	1/1	1/1	0
Injection site erythema	1/1	0	1/1	0	0	0	0
Infusion site extravasation	1/1	0	0	0	0	2/2	0
Injection site extravasation	0	1/1	1/1	0	0	3/4	0
Injection site hemorrhage	0	0	2/2	0	0	0	0
Injection site injury	0	0	2/2	0	0	0	0
Vessel puncture site hemorrhage	0	0	2/2	0	0	0	0
Headache	1/1	0	1/1	0	0	3/3	0
Ecchymosis	1/1	0	0	0	0	2/3	0
Scratch	0	0	1/1	0	1/1	0	0
Phlebitis	0	0	0	0	0	4/9	0
Nausea	0	0	0	0	0	3/4	0
Diarrhea	0	0	0	0	0	2/3	0

a AE, adverse event; E, number of TEAEs; ECG, electrocardiogram; MAD, multiple ascending dose; *n*, number of participants with a TEAE; TEAE, treatment-emergent adverse event.

b Erythema at the ECG patch sites.

Nine of the 11 (81.8%) participants who received nacubactam coadministered with meropenem during part 3 of the MAD study reported a total of 42 AEs (Table 2), which was consistent with the known safety profile of meropenem. The most common AEs were phlebitis (36.7%), injection site extravasation (27.3%), headache (27.3%), and nausea (27.3%). Overall, the safety profile was similar in participants who received nacubactam plus meropenem compared with those who received nacubactam alone. Among the four participants who received placebo in part 3 of the MAD study, one (25.0%) participant reported an AE.

In part 3 of the MAD study, one participant withdrew from the study due to mild to moderate AEs after receiving a single dose of nacubactam of 2,000 mg (day 1), a single dose of meropenem of 2,000 mg (day 2), a single dose of nacubactam of 2,000 mg in combination with meropenem of 2,000 mg on day 3, and 11 doses of nacubactam coadministered with meropenem (days 4 to 7); the reported AEs included mild diarrhea and nausea and moderate infusion site extravasation.

Overall, there was no apparent relationship between drug dose and the pattern, incidence, or severity of AEs. Furthermore, no clinically relevant dose-related trends were observed in any of the safety parameters monitored (i.e., clinical laboratory tests, vital signs, and electrocardiogram [ECG]). No serious AEs, dose-limiting AEs, or deaths were reported in either study.

No participant had clinically relevant changes or apparent dose-related trends in urinalysis parameters or validated serum measures of renal function, including serum creatinine, cystatin C, blood urea nitrogen, and electrolytes. There were no marked changes in exploratory urinary kidney biomarkers, namely, microalbumin, kidney injury molecule-1, and *N*-acetyl-β-d-glucosaminidase. There were no apparent dose-related trends in the average absolute values or changes from baseline of these exploratory markers, and out-of-reference-range values showed no apparent relationship to nacubactam dose. However, concentrations of these exploratory biomarkers tended to be highest at the end of the treatment period in participants who received 2,000 mg nacubactam coadministered with 2,000 mg meropenem. While the majority of changes were small and not consistent across different biomarkers, one participant had concomitantly larger changes in all three markers; all changes were transient and reversible after the end of dosing, and there was no evidence of clinically relevant renal injury.

### Pharmacokinetics.

Following a single i.v. administration, plasma concentrations of nacubactam peaked at the end of the infusion, followed by a rapid monophasic decrease in plasma levels. Nacubactam exposure (maximum observed plasma concentration and area under the curve [*C*_max_/AUC]) increased in an approximately dose-proportional manner (see Fig. S1 in the supplemental material). Total clearance (CL) and volume of distribution at steady state (*V*_ss_) were constant, with mean CL values ranging from 7.03 to 9.87 liters/h and mean *V*_ss_ values ranging from 17.03 to 22.40 liters across the doses examined (Table 3). Renal clearance (CL_R_) remained consistent with increasing nacubactam dose, with the mean CL_R_ ranging from 6.86 ± 0.64 liters/h to 9.66 ± 1.66 liters/h over the five dose levels. The mean apparent terminal elimination half-life (*t*_1/2_) ranged from 1.76 ± 0.17 h to 2.36 ± 0.46 h across the doses examined.

**TABLE 3:** Summary of single-dose nacubactam pharmacokinetics in the SAD study

Parameter^*a*^	Value(s) for nacubactam dose of^*b*^:				
	50 mg (*n* = 6)	150 mg (*n* = 6)	500 mg (*n* = 6)	1,000 mg (*n* = 6)	2,000 mg (*n* = 6)
*C*_max_ (μg/ml)	2.69 (0.91)	9.02 (1.47)	31.28 (4.49)	72.05 (10.25)	112.15 (21.85)
*t*_max_ (h)	0.54 (0.10)	0.50 (0.00)	0.50 (0.00)	0.50 (0.00)	0.50 (0.00)
AUC_0–last_ (h·μg/ml)	5.08 (0.96)	16.12 (2.43)	53.55 (5.02)	142.38 (12.14)	228.98 (19.87)
AUC_0–inf_ (h·μg/ml)	5.21 (0.95)	16.26 (2.42)	53.87 (5.03)	143.23 (12.25)	229.20 (19.89)
*t*_1/2_ (h)	1.83 (0.22)	1.76 (0.17)	1.77 (0.15)	2.05 (0.37)	2.36 (0.46)
*k*_el_ (1/h)	0.38 (0.05)	0.40 (0.04)	0.39 (0.04)	0.35 (0.06)	0.31 (0.08)
CL (liters/h)	9.87 (1.86)	9.40 (1.43)	9.35 (0.88)	7.03 (0.61)	8.78 (0.71)
*V*_ss_ (liters)	22.40 (4.88)	20.23 (3.18)	19.55 (2.09)	17.03 (2.15)	20.56 (3.55)

a AUC_0–inf_, area under the plasma concentration-time curve from time zero to infinity; AUC_0–last_, area under the plasma concentration-time curve from time zero until the last quantifiable time point; CL, clearance; *C*_max_, maximum observed plasma concentration; *k*_el_, apparent terminal elimination rate constant; SAD, single ascending dose; *t*_1/2_, apparent terminal elimination half-life; *t*_max_, time to reach maximum plasma concentration; *V*_ss_, volume of distribution at steady state.

b Data are presented as means (standard deviations).

**FIG 2:**
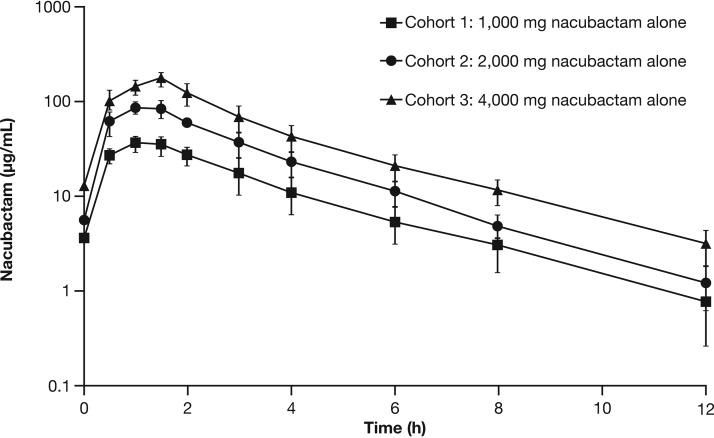
Log-linear overlay plot of mean nacubactam plasma concentrations over time after nacubactam q8h dosing for 7 days in the MAD study. Data are arithmetic means ± standard deviations from day 7 (part 1) or day 9 (part 2).

Following multiple i.v. doses of nacubactam, peak and total exposures (i.e., *C*_max_ and AUC from time zero to 8 h [AUC_0–8h_]) increased in a dose-proportional manner (Fig. 2). Analysis of variance (ANOVA) comparisons of dose-normalized nacubactam exposure parameters between pairs of doses revealed no consistent departures from dose proportionality across the dose range. There was no significant change in CL, CL_R_, *V*_ss_, or *t*_1/2_ across the examined dose range following a single dose or repeat q8h dosing (Table 4). Variability in all pharmacokinetic parameters was generally low (coefficient of variation of <27%) and did not appear to change across the dose range. There was minimal accumulation of nacubactam in the plasma following 7 days of q8h dosing.

**TABLE 4:** Summary of pharmacokinetics of nacubactam in the MAD study^*d*^

Parameter^*c*^	Part 1^*a*^		Part 2^*a*^^,^^*b*^	Part 3	
	Nacubactam, 1,000 mg (*n* = 6)	Nacubactam, 2,000 mg (*n* = 6)	Nacubactam, 4,000 mg (*n* = 6)	Single dose (day 1 or 2) nacubactam, 2,000 mg	Single dose (day 3) nacubactam, 2,000 mg, plus meropenem, 2,000 mg
*C*_max_ (μg/ml)	36.6 (21)	89.6 (15)	179 (17)	66.0 (14)	65.0 (30)
*t*_max_ (h)	1.00 (1.00 to 1.67)	1.33 (0.90 to 1.67)	1.50 (1.00 to 1.53)	1.00 (1.00 to 1.67)	1.00 (1.00 to 1.67)
AUC_0–8h_ (μg·h/ml)	118 (23)	279 (13)	496 (18)	201 (15)	204 (27)
AUC_0–inf_ (μg·h/ml)	NA	NA	NA	225 (18)	224 (30)
*t*_1/2_ (h)	2.66 (5)	2.66 (5)	2.69 (5)	2.63 (30)	2.38 (14)
CL (liters/h)	8.50 (23)	7.16 (13)	8.06 (18)	8.87 (18)	8.93 (30)
CL_R_ (liters/h)	6.69 (27)	6.23 (19)	6.77 (19)	8.52 (18)	8.10 (32)
*V*_ss_ (liters)	21.9 (17)	16.5 (13)	20.1 (23)	26.2 (33)	25.8 (30)
Fe%_0–8h_	81.9 (1)	87.0 (11)	83.9 (9)	85.5 (13)	82.7 (12)
Fe%_0–24h_	NA	NA	NA	91.1 (10)	88.4 (11)
*R*_ac_ (*C*_max_)	1.04 (8)	1.08 (18)	1.02 (18)	NR	NR
*R*_ac_ (AUC_0–8h_)	1.107 (8)	1.06 (10)	1.02 (7)	NR	NR

a q8h administration for 7 days. Data are from day 7 (part 1) or day 9 (part 2).

b Cohort 4 excluded was from the table due to being single-dose administration only.

c AUC_0–inf_, area under the plasma concentration-time curve from time zero to infinity; AUC_0–8h_, area under the plasma concentration-time curve from time zero to 8 h; CL, clearance; CL_R_, renal clearance; *C*_max_, maximum observed plasma concentration; Fe%_0–8h_, cumulative percentage of dose excreted in urine from time zero to 8 h; Fe%_0–24h_, cumulative percentage of dose excreted in urine from time zero to 24 h; MAD, multiple ascending dose; *R*_ac_ (*C*_max_), accumulation ratio based on *C*_max_; *R*_ac_ (AUC_0–8h_), accumulation ratio based on AUC_0–8h_; *t*_1/2_, apparent terminal elimination half-life; *t*_max_, time to reach maximum plasma concentration; *V*_ss_, volume of distribution at steady state.

d Data presented as geometric means and percent coefficient of variation, except *t*_max_, which is given as medians (ranges). NA, not applicable; NR, not reported.

**FIG 3:**
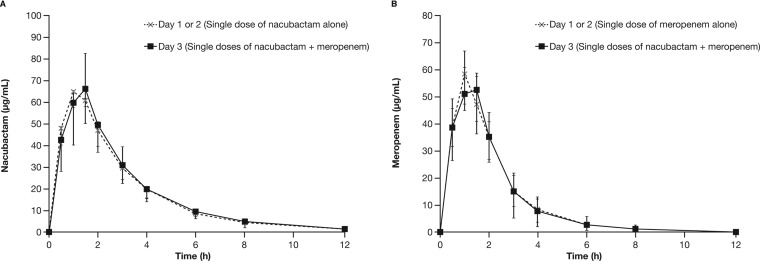
Mean nacubactam (A) and meropenem (B) plasma concentration-time profiles after dosing with nacubactam alone or nacubactam coadministered with meropenem.

A summary of nacubactam and meropenem pharmacokinetic parameters after dosing alone or in combination is shown in Table 4 and Table S2 in the supplemental material, respectively. Single-dose pharmacokinetic parameters of nacubactam were similar when nacubactam was administered alone or coadministered with meropenem (Fig. 3A). The 90% confidence intervals (CIs) of the geometric least square mean ratios of the *C*_max_, AUC, CL, *V*_ss_, and CL_R_ of nacubactam between monotherapy and in combination with meropenem were fully contained within the equivalence limits of 0.80 to 1.25 (CL geometric mean ratio [GMR], 1.01; 90% CI, 0.87 to 1.16; *V*_ss_ GMR, 0.98; 90% CI, 0.87 to 1.11; CL_R_ GMR, 0.95; 90% CI, 0.85 to 1.07).

Single-dose pharmacokinetic parameters of meropenem were also similar when meropenem was administered alone or with nacubactam (Fig. 3B); 90% CIs for the geometric least square mean ratios for comparison between treatments for the meropenem parameters CL, *V*_ss_, and CL_R_ were fully contained within the equivalence limits of 0.80 to 1.25 (CL GMR, 0.99; 90% CI, 0.94 to 1.03; *V*_ss_ GMR, 1.09; 90% CI, 1.01 to 1.18; CL_R_ GMR, 0.95; 90% CI, 0.87 to 1.05). While initial plasma concentrations of the two agents administered as 2,000-mg doses were similar (i.e., meropenem/nacubactam ratio of approximately 0.7), meropenem concentrations were markedly lower than corresponding nacubactam concentrations at the end of the dosing interval (q8h over 6 days, i.e., ratio of <0.2).

### Nacubactam metabolism.

Following single and multiple doses, nacubactam was primarily eliminated through direct renal excretion; the majority of the nacubactam dose was excreted largely unchanged into urine, with minimal metabolic clearance.

Metabolite profiling did not identify any major metabolites. Two minor metabolites, RO7110880 (open ring analog, M1) and RO7053802 (deaminated ethoxy analog, M2), which were both previously identified in animal studies, were present at exposures of ∼4% and <1% of corresponding nacubactam exposures, respectively. The respective mean (percent coefficient of variation) AUC_0–inf_ after multiple doses of nacubactam at 1,000 mg, 2,000 mg, and 4,000 mg were 5.72 (39%), 12.4 (22%), and 24.9 (19%) μg·h/ml, respectively, for RO7110880 and 0.290 (33%), 0.366 (24%), and 0.411 (40%) μg·h/ml, respectively, for RO7053802.

A covalent nacubactam-meropenem adduct (RO7120597), which had been identified previously in *in vitro* studies, was detectable at low concentrations in plasma after dosing of nacubactam coadministered with meropenem. After adjusting for molecular weight, RO7120597 exposures were <1/1,500 the corresponding nacubactam and meropenem exposures. Approximately 11 mg and 18 mg of RO7120597 was excreted in urine on days 3 and 9, respectively, representing approximately 0.26% and 0.42% of the administered nacubactam dose, respectively.

### ECG findings.

**FIG 4:**
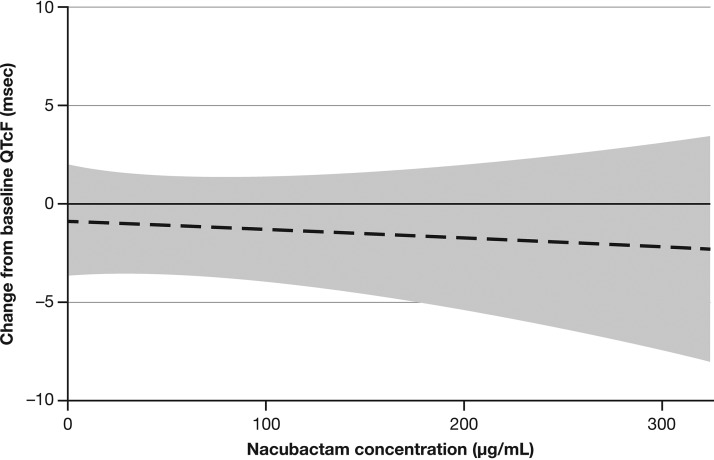
Placebo-adjusted change in QTcF as a function of concentration (with 90% confidence intervals) from linear model.

Intensive ECG monitoring did not reveal any significant effects of nacubactam dosing on the heart rate-corrected QT (QTc) interval duration, other ECG intervals, or ECG morphology. A prespecified exposure-response analysis revealed no relationship between nacubactam plasma concentrations and changes in QTc interval using Fridericia’s formula (QTcF); predictions indicated that a QTcF prolongation of 10 ms could be excluded across the range of observed plasma exposures. The linear model fit the data adequately; the residuals appeared randomly and independently distributed across the range of predicted values, and no significant departure from normality was apparent. The slope, intercept, and all other terms of the linear model were not statistically significant (slope estimate, 0.00422; 90% CI, 0.02509 to 0.01665; *P* = 0.73; treatment estimate, 0.9474; 90% CI, 3.8879 to 1.9931; *P* = 0.59). Figure 4 shows the placebo-adjusted change in QTcF as a function of concentration based on the linear model. Quadratic and log-linear models did not provide a significantly better fit to the data. For each of the three models, the predicted mean change from baseline in QTcF at the *C*_max_ of 325 μg/ml was <3 ms, while the upper limit of the two-sided 90% CI was <10 ms.

Nacubactam coadministered with meropenem also had no apparent effect on QTc interval duration, other ECG intervals, or ECG morphology.

## DISCUSSION

This paper reports the results from two phase I studies that evaluated the pharmacokinetics and safety of single and multiple escalating doses of nacubactam in healthy volunteers, including the assessment of nacubactam administered in combination with meropenem.

Nacubactam administered alone (50 to 8,000 mg) or coadministered with meropenem (nacubactam at 2,000 mg with meropenem at 2,000 mg) was generally well tolerated, and no serious AEs were reported. No significant effect of nacubactam dosing on QT interval duration or other ECG parameters was observed. The assessment of the potential for a new chemical entity to cause QT interval prolongation is an integral part of the clinical development program, and regulatory guidelines often recommend a dedicated clinical study to evaluate drug effects on the QT interval (termed a thorough QT study). However, recent publications support the use of pharmacokinetic/pharmacodynamic modeling of data from other studies to inform about the risk of QT prolongation (4–6). To this end, a prespecified exposure-response analysis was performed using a mixed-effects linear model. This analysis revealed no significant relationship between the plasma concentration of nacubactam and change in QTcF from the baseline, and that QTcF prolongation of 10 ms could be excluded across the range of observed plasma exposures. These findings support the conclusion that the therapeutic administration of nacubactam is not expected to have any clinically important effects on cardiac repolarization.

Nacubactam had no significant effects on kidney function; there were no clinically relevant changes or dose-related trends in validated measures of renal function or exploratory urine kidney biomarkers among participants who received nacubactam alone. However, concentrations of exploratory urinary kidney biomarkers (microalbumin, kidney injury molecule-1, and *N*-acetyl-β-d-glucosaminidase) tended to be highest after treatment with 2,000 mg nacubactam coadministered with 2,000 mg meropenem. The use of β-lactam antibiotics has been associated with nephrotoxicity, including acute interstitial nephritis, glomerulonephritis, and acute tubular necrosis (7–9). Therefore, these changes could be attributable to concomitant dosing of meropenem; however, the interpretation of these findings was confounded by the high variability in levels of the markers in all groups (including placebo) and the absence of an appropriate control group (i.e., meropenem alone).

The pharmacokinetics of nacubactam following single (1,000 to 8,000 mg) and multiple (1,000 to 4,000 mg) doses were linear, and primary pharmacokinetic parameters were not affected by the length of infusion (i.e., 30 versus 90 min). No differences in pharmacokinetic profiles were observed when nacubactam was administered alone or in combination with meropenem. The majority of the administered nacubactam dose was excreted unchanged via the kidneys, which suggests that kidney function is the principle factor influencing nacubactam exposure and that clinical dosing will have to account for the renal function of recipients. The estimated CL of nacubactam ranged from 7.2 to 8.9 liters/h on average, while the estimated CL_R_ ranged from 5.5 to 8.5 liters/h. This CL_R_ range is also similar to the estimated creatinine clearance derived from serum creatinine concentrations at baseline (approximately 120 ml/min or 7.2 liters/h on average). Direct comparison is possible because nacubactam is minimally protein bound (fraction unbound, 0.98, i.e., 2% protein binding [unpublished data]). These results indicate that nacubactam is cleared almost completely by glomerular filtration with no significant net tubular secretion. Furthermore, the small difference between CL and CL_R_ indicates a minor contribution of nonrenal elimination processes on clearance (i.e., <20%); this is consistent with the observation that metabolites M1 and M2 were formed in small amounts.

The results reported here demonstrate that the pharmacokinetic profile of nacubactam is similar to that of other β-lactamase inhibitors. Pharmacokinetic studies of avibactam, relebactam, and vaborbactam in healthy individuals showed that the drug exposure increased in a dose-proportional manner, with approximately 2-h half-lives, and that the drugs were excreted largely unchanged in urine (10–13). Moreover, nacubactam pharmacokinetics are also similar to those of meropenem, thereby confirming its potential suitability as a partner for β-lactam antibiotics.

Plasma exposures and cumulative urinary excretion of meropenem in the present study were consistent with those typically observed with meropenem when administered alone (14). There were no differences in the pharmacokinetics of meropenem when given alone or coadministered with nacubactam. This suggests that the established meropenem pharmacokinetic models and clinical dosing algorithms based on renal function (and bodyweight for pediatric patients) remain appropriate for dose recommendations of meropenem when coadministered with nacubactam.

A covalent nacubactam-meropenem adduct (RO7120597) was detected in low concentrations in the plasma when nacubactam and meropenem were coadministered. This adduct had been previously identified in *in vitro* studies of nacubactam and meropenem: when infusion solutions were mixed at room temperature, the adduct formed in a concentration-dependent manner (approximately 3% using starting drug concentrations of 10 mg/ml; unpublished data). In this study, the two drugs were administered separately via a dual-lumen catheter to avoid the formation of the adduct before entry into the body and thereby allow unambiguous assessment of adduct formation *in vivo*. Only very low concentrations of adduct were detected in plasma. While the adduct was detected at a higher concentration in the urine, this was presumably as a result of the continued formation of the adduct during the retention of urine in the bladder and during urine sample collection, storage, and processing.

To conclude, nacubactam, alone or coadministered with meropenem, was well tolerated by healthy volunteers, and no relevant safety signals were identified. No significant effect on QT interval duration or other ECG parameters was observed. The pharmacokinetics of nacubactam is linear and predictable, and the drug is primarily excreted unchanged via the urine. The favorable safety and pharmacokinetic profile of nacubactam shown in these studies support continued clinical development. Furthermore, these results provide a robust basis for nacubactam dose selection during later stages of clinical development. The pharmacokinetic characteristics of nacubactam and the absence of drug-drug interactions with meropenem support the use of this carbapenem as a β-lactam partner for nacubactam.

## MATERIALS AND METHODS

### Study participants.

The SAD study (ClinicalTrials registration no. NCT02134834) enrolled healthy Caucasian males, aged 18 to 45 years, with a body mass index of 18.0 to 30.0 kg/m^2^ at screening. The MAD study (ClinicalTrials registration no. NCT02972255) included healthy males or females (of non-childbearing potential), aged 18 to 70 years. The full exclusion criteria for each study are provided in the supplemental material.

### Study design and treatments.

The two studies were randomized, double-blind, placebo-controlled studies that enrolled participants into sequential treatment cohorts using a 3:1 active treatment-to-placebo randomization ratio. In the SAD study, following a 27-day screening period, participants were enrolled into five sequential treatment cohorts of ascending nacubactam doses (50 mg, 150 mg, 500 mg, 1,000 mg, and 2,000 mg) or placebo (Fig. 1).

The MAD study consisted of three parts (Fig. 1). Part 1 assessed lower doses of nacubactam. Participants were enrolled in two sequential cohorts (cohort 1, followed by cohort 2). In each cohort, participants were randomized to receive either nacubactam or placebo q8h for 7 days. The doses of nacubactam were 1,000 mg in cohort 1 and 2,000 mg in cohort 2.

Part 2 assessed higher doses of nacubactam not previously studied when given as single doses. Participants were also enrolled in two sequential cohorts (cohort 3, followed by cohort 4). Cohort 3 received single-dose nacubactam at 4,000 mg or placebo on day 1, extending to q8h dosing for 7 days once safety and tolerability were confirmed 48 h after the single dose. Cohort 4 received a single dose of 8,000 mg nacubactam.

Part 3 of the MAD study randomized participants to nacubactam coadministered with meropenem or placebo. In the active treatment group, participants were further randomized 1:1 to receive one of two treatment sequences on days 1 and 2, either 2,000 mg nacubactam alone on day 1 and 2,000 mg meropenem alone on day 2 or *vice versa*. All participants in the active group received a single 2,000-mg dose of both drugs on day 3 and q8h on days 4 to 8, with a final single dose of both drugs on the morning of day 9. A washout period of approximately 24 h between treatments was provided.

Study treatments were administered as 30-min (SAD study) or 90-min (MAD study) i.v. infusions, the latter to investigate the pharmacokinetics using a longer infusion time, which is commonly used to increase the time above the MIC of β-lactams (15). The choice of a 90-min infusion time for the MAD study was dictated by the stability and toxicology data available at the time the study was being designed. The coadministration of nacubactam and meropenem was performed via a dual-lumen catheter to avoid the formation of an adduct between the two drugs during infusion.

A sentinel dosing strategy was employed in both studies. For the SAD study, two participants in cohort 1 (one for 50 mg nacubactam and one for placebo) were dosed 24 h prior to the remaining participants to allow for a mandatory safety review of these two sentinel participants. In all cohorts, escalation to the next dose occurred after a safety review of the previous cohort. In the MAD study, sentinel dosing was performed in part 3, wherein participants were evaluated for safety up to 24 h after administration of the first drug combination (or placebo) on day 3, prior to enrolling additional participants.

### Study assessments.

Safety assessments included monitoring of AEs, laboratory safety tests (hematology, serum chemistry, and urinalysis), 12-lead ECG (discrete recordings and 24-h Holter recording), and vital signs (blood pressure, pulse rate, and body temperature). In the SAD study, safety assessments were performed predose at screening, at day 1, postdose periodically on days 1, 2, and 7 (follow-up), and at early withdrawal, if relevant. Blood samples for pharmacokinetic profiling in the SAD study were collected on day 1 before the morning infusion and 0.5, 1, 1.5, 2, 3, 4, 6, 8, 12, and 24 h postinfusion start. In the MAD study, safety assessments were done predose at screening, at day 2, postdose periodically on days 1, 2, 4, 7, and 8, on days 13 to 15 (follow-up), and at early withdrawal, if relevant. In the MAD study, blood samples were collected on days 1 and 9 prior to the morning infusion and 0.5, 1, 1.5, 2, 3, 4, 6, 8, and 12 h (on day 9 only) postinfusion start. On all other days, a single predose sample was collected for pharmacokinetic evaluation prior to the morning infusion. Blood was collected into lithium-heparin tubes and stored at a nominal –80°C temperature until analysis.

### Bioanalytical assays.

Plasma and urine concentrations of nacubactam, meropenem, and RO7120597 (nacubactam-meropenem adduct) were determined by specific, validated liquid chromatography tandem mass spectrometry assays (assay performance data during sample analysis are given in Tables S3 and S4 in the supplemental material). Metabolite profiling and plasma and urine concentrations of nacubactam metabolites were also determined by liquid chromatography tandem mass spectrometry, with a lower limit of quantification of 10.0 ng/ml in plasma and 50.0 ng/ml in urine.

### Statistical analyses.

The sample sizes for these studies were selected without performing a power calculation to provide descriptive information on safety, tolerability, and pharmacokinetics.

All pharmacokinetic parameters (CL, CL_R_, *V*_ss_, AUC_0–8h_, AUC_0–inf_, Fe%_0–8h_, Fe%_0–24h_, *C*_max_, *t*_max_, *t*_1/2_, *R*_ac_ [*C*_max_] [i.e., accumulation ratio based on *C*_max_], *R*_ac_ [AUC_0–8h_], *k*_el_, and *V*_ss_) were determined using standard noncompartmental methods using Phoenix WinNonlin, v.6.3 and higher (Certara Inc., Princeton, NJ, USA).

Mean plasma concentrations and pharmacokinetic parameters of nacubactam (plus its open ring [M1, RO7110880] and deaminated ethoxy analog [M2, RO7053802] metabolites) and meropenem were summarized, and descriptive statistics were applied to the data.

An exploratory evaluation of dose proportionality was performed by ANOVA with selected pharmacokinetic parameters normalized by dose.

A mixed-effects repeated-measures linear model was used to quantify the potential relationship between nacubactam plasma concentrations and QTcF. Nacubactam plasma concentration was the continuous independent variable, change from baseline in QTcF was the dependent variable, and time point and treatment (nacubactam or placebo) were fixed effects.

Drug-drug interactions between nacubactam and meropenem were analyzed using an analysis of covariance (ANCOVA) linear mixed-model analysis applied to the log-transformed primary pharmacokinetic parameters (*C*_max_, AUC, CL, *V*_ss,_ and CL_R_).

### Ethical considerations.

The study protocols for the SAD and MAD studies were approved by applicable ethics committees and institutional review boards, and the studies were conducted in accordance with the Declaration of Helsinki and Good Clinical Practice. Written informed consent was obtained from the study participants prior to study initiation.

### Data availability.

Qualified researchers may request access to individual patient-level data through the clinical study data request platform (www.clinicalstudydatarequest.com). Further details on Roche’s criteria for eligible studies are available at https://clinicalstudydatarequest.com/Study-Sponsors/Study-Sponsors-Roche.aspx. For further details on Roche’s global policy on the sharing of clinical information and how to request access to related clinical study documents, see https://www.roche.com/research_and_development/who_we_are_how_we_work/clinical_trials/our_commitment_to_data_sharing.htm.

## Supplementary Material

Supplemental file 1
