# Supplementary material for: Safety and Pharmacokinetic Characterization of Nacubactam, a Novel β-Lactamase Inhibitor, Alone and in Combination with Meropenem, in Healthy Volunteers
Source: Antimicrob Agents Chemother. 2020 Apr 21;64(5):e02229-19. doi: 10.1128/AAC.02229-19 (PMC7179653; doi:10.1128/AAC.02229-19)

**SUPPLEMENTARY APPENDIX**

**Exclusion criteria.** Exclusion criteria for the single ascending dose (SAD) study included: receipt of any investigational agent or drug within 4 months before screening; a history or current evidence of allergic symptoms (such as bronchial asthma, drug-induced rash, or urticarial); hypersensitivity and/or allergy to drugs, (including β-lactam agents); concurrent or history of clinically significant cardiovascular, hepatic, renal, endocrine, gastrointestinal, respiratory, psychiatric, neurologic and/or hematologic disorders; QT intervals of 450 ms or longer from 12-lead electrocardiogram (ECG); concurrent or history of hypotension (blood pressure [BP] 90/50 mmHg or less) or hypertension (BP 150/90 mmHg or more); a history of chronic or recurrent infections or current active infection; a history or presence of malignancy (with the exception of successfully treated basal cell carcinoma); presence of current infection with hepatitis B surface antigen, hepatitis C virus antibody, or human immunodeficiency virus; donation of blood (or loss of blood) >400 mL within 3 months before screening; consumption of 21 units or more of alcoholic beverages per week or inability to abstain from alcohol during the study period; a history of smoking at any time within 1 year before screening; inability or unwillingness to use an adequate form of contraception for 3 months following the study.

In addition to the criteria above, the multiple ascending dose (MAD) study also included the following exclusion criteria: pregnant or lactating women; known hypersensitivity and/or allergy to any component of meropenem (part 3 only) or to other drugs in the same class; history of any severe antibiotic-associated superinfections, such as *Clostridium difficile* colitis and/or frequent fungal vaginal infections; a history of

seizures, brain lesions, or other significant neurologic diseases; personal history of congenital long QT syndrome or family history of sudden death; history of Gilbert syndrome; healthy volunteers who had significant acute infection, e.g., influenza, local infection, acute gastrointestinal symptoms, or any other clinically significant illness within 2 weeks of dose administration; clinically relevant ECG abnormalities at screening, e.g., heart rate-corrected QT interval using the Fridericia's formula >450 ms or <300 ms, notable resting bradycardia (heart rate <45 beats per min), or heart rate >90 beats per min, ECGs with documented machine errors in the interval duration assessments, evidence of atrial fibrillation, atrial flutter, complete bundle branch block, Wolf-Parkinson-White Syndrome, or cardiac pacemaker; ECG with QRS and/or T-wave judged to be unfavorable for a consistently accurate QT measurement; creatinine clearance <70 mL/min (using the Cockcroft-Gault formula); coffee (or tea) consumption of >5 cups/day or >1.5 L/day methylxanthine-containing drinks, or >250 g/day of chocolate.

**TABLE S1** Summary of TEAEs by treatment group in the SAD study

| | Nacubactam 50 mg (*n* = 6) | Nacubactam 150 mg (*n* = 6) | Nacubactam 500 mg (*n* = 6) | Nacubactam 1,000 mg (*n* = 6) | Nacubactam 2,000 mg (*n* = 6) | Placebo (*n* = 10) |
|---|---|---|---|---|---|---|
| ≥1 TEAE (*n*/E) | 2/3 | 1/3 | 2/2 | 2/2 | 1/1 | 4/5 |
| ≥1 severe TEAE (*n*) | 0 | 0 | 0 | 0 | 0 | 0 |
| ≥1 drug-related TEAE (*n*/E) | 0 | 0 | 0 | 0 | 1/1 | 2/2 |
| ≥1 serious TEAE (*n*) | 0 | 0 | 0 | 0 | 0 | 0 |
| Specific TEAEs (*n*/E) | | | | | | |
| Headache | 0 | 0 | 1/1 | 0 | 1/1 | 3/3 |
| Application site rash | 0 | 0 | 0 | 0 | 0 | 2/2 |
| Chest discomfort | 1/1 | 0 | 0 | 0 | 0 | 0 |
| Fatigue | 1/1 | 0 | 0 | 1/1 | 0 | 0 |
| Feeling hot | 0 | 1/1 | 0 | 0 | 0 | 0 |
| Cough | 0 | 1/1 | 0 | 0 | 0 | 0 |
| Nasal congestion | 1/1 | 0 | 0 | 0 | 0 | 0 |

| | | | | | | |
|---|---|---|---|---|---|---|
| Sinus tachycardia | 0 | 1/1 | 0 | 0 | 0 | 0 |
| Abdominal pain | 0 | 0 | 0 | 1/1 | 0 | 0 |
| Rhinitis | 0 | 0 | 1/1 | 0 | 0 | 0 |

E, number of TEAEs; *n*, number of participants with a TEAE; SAD, single ascending dose; TEAE, treatment-emergent adverse event.

**TABLE S2** Summary of meropenem pharmacokinetics after dosing alone or with nacubactam (prt 3)

| Parameter | Single dose (day 1 or 2) meropenem 2,000 mg | Single dose (day 3) nacubactam 2,000 mg + meropenem 2,000 mg | Repeat dosing (day 9) nacubactam 2,000 mg + meropenem 2,000 mg |
|---|---|---|---|
| $C_{max}$ (µg/mL) | 57.8 (20) | 54.3 (22) | 53.9 (16) |
| $t_{max}$ (h) | 1.0 (1.0 to 1.0) | 1.0 (1.0 to 1.7) | 1.0 (0.6 to 1.7) |
| $AUC_{0–8h}$ (µg•h/mL) | 130 (27) | 131 (26) | 126 (30) |
| $AUC_{0–inf}$ (µg•h/mL) | 132 (29) | 133 (27) | NA |
| $t_{1/2}$ (h) | 1.3 (35) | 1.2 (20) | 1.3 (24) |
| CL (L/h) | 15.2 (29) | 15.0 (27) | 15.9 (30) |
| $CL_r$ (L/h) | 9.68 (35) | 9.24 (30) | 9.91 (35) |
| $Vd_{ss}$ (L) | 19.8 (18) | 21.5 (26) | 20.0 (14) |
| $Fe\%_{0–8h}$ | 62.7 (14) | 60.6 (16) | 62.3 (15) |
| $Fe\%_{0–24h}$ | 63.7 (13) | 61.5 (16) | NA |
| $Ae_{0–8h}$ (mg) | 1,250 (14) | 1,210 (16) | 1,240 (15) |
| $Ae_{0–24h}$ (mg) | 1,270 (13) | 1,230 (16) | NA |

Data presented as geometric means and % coefficient of variation, except $t_{max}$ which is median (range).

$Ae_{0–8h}$, cumulative amount excreted in urine from time 0 to 8 h; $Ae_{0–24h}$, cumulative amount excreted in urine from time 0 to 24 h; $AUC_{0–8h}$, area under the plasma concentration-time curve from time 0 to 8 h; $AUC_{0–inf}$, area under the plasma concentration–time curve from time 0 to infinity; CL, total clearance; $CL_r$, renal clearance; $C_{max}$, maximum observed plasma concentration; $Fe\%_{0–8h}$, cumulative percent of dose excreted in urine from time 0 to 8 h; $Fe\%_{0–24h}$, cumulative percent of dose excreted in urine from time 0 to 24 h; NA, not applicable; $t_{1/2}$, apparent terminal elimination half-life; $t_{max}$, time to reach maximum plasma concentration; $Vd_{ss}$, volume of distribution at steady state.

**TABLE S3.** Summary of the validated LC-MS/MS assay performance for measurement of PK samples of the SAD study

| Analyte | Matrix | Linear (Calibration) range | Individual QC accuracy, over all QC levels (%) |
|---|---|---|---|
| Nacubactam | Plasma | 0.02 to 5 µg/mL | 89.1 to 119.2 |
| RO7110880 | Plasma | 0.02 to 5 µg/mL | 87.3 to 117.8 |
| RO7053802 | Plasma | 0.02 to 2 µg/mL | 88.4 to 115.7 |

LC-MS/MS, liquid chromatography-mass spectrometry; SAD, single ascending dose; PK, pharmacokinetic; QC, quality control sample

**TABLE S4.** Summary of the validated LC-MS/MS assay performance for measurement of PK samples of the MAD study

| Analyte | Matrix | Linear (calibration) range | QC levels overall precision (RSD%) | QC levels overall accuracy (%) |
|---|---|---|---|---|
| Nacubactam | Plasma | 10.0 to 10000 ng/mL | 4.1 to 9.4 | 97.4 to 103.7 |
| Meropenem | Plasma | 0.100 to 100 µg/mL | 2.9 to 7.6 | 98.4 to 101.3 |
| RO7120597 | Plasma | 5.00 to 5000 ng/mL | 4.1 to 11.4 | 100.5 to 105.1 |
| Nacubactam | Urine | 50.0 to 50000 ng/mL | 4.5 to 6.3 | 96.5 to 97.7 |
| Meropenem | Urine | 10.0 to 10000 µg/mL | 3.6 to 12.1 | 98.0 to 109.3 |
| RO7120597 | Urine | 20.0 to 20000 ng/mL | 2.7 to 4.1 | 99.4 to 101.8 |

LC-MS/MS, liquid chromatography-mass spectrometry; MAD, multiple ascending dose; PK, pharmacokinetic; QC, quality control sample; RSD%, relative standard deviation.

**FIG S1** Log-linear overlay plot of mean nacubactam plasma concentration over time after a single dose of nacubactam.

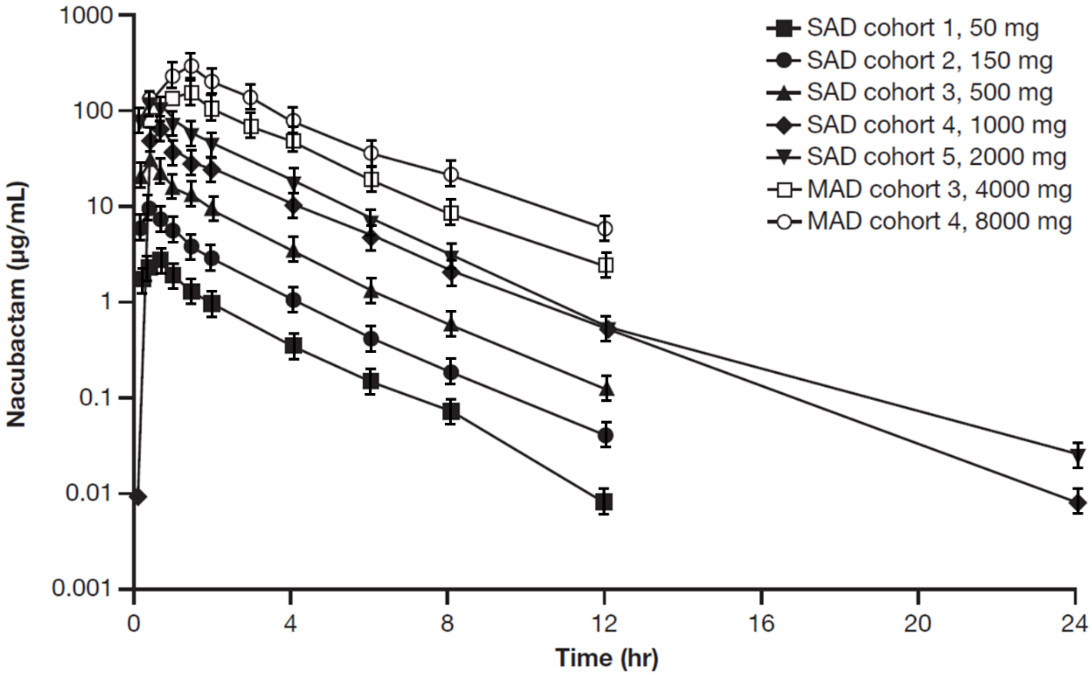

Supplement: Supplemental file 1 [file AAC.02229-19-s0001.pdf]
